# 2199. Herpesviridae lung reactivation and infection in patients with severe Covid-19 or influenza virus pneumonia: a comparative study

**DOI:** 10.1093/ofid/ofac492.1818

**Published:** 2022-12-15

**Authors:** Charles-Edouard Luyt, Sonia Burrel, Vincent Guiraud, Alain Combes, David Boutolleau

**Affiliations:** Assistance publique hopitaux de Paris, Paris, Ile-de-France, France; Assistance publique hopitaux de Paris, Paris, Ile-de-France, France; Assistance publique hopitaux de Paris, Paris, Ile-de-France, France; Assistance publique hopitaux de Paris, Paris, Ile-de-France, France; Assistance publique hopitaux de Paris, Paris, Ile-de-France, France

## Abstract

**Background:**

Lung reactivations of *Herpesviridae*, herpes simplex virus (HSV) and cytomegalovirus (CMV) have been reported in Covid-19 patients. Whether or not those viral reactivations are more frequent than in other patients is not known.

**Methods:**

Retrospective monocentric cohort study of 145 patients with severe Covid-19 pneumonia requiring invasive mechanical ventilation and who were tested for HSV and CMV in bronchoalveolar lavage performed during fiberoptic bronchoscopy for ventilator-associated pneumonia suspicion. Rates of HSV and CMV lung reactivations, and HSV bronchopneumonitis were assessed and compared with an historical cohort of 89 patients with severe influenza pneumonia requiring invasive mechanical ventilation.

**Results:**

Among the 145 Covid-19 patients included, 50% and 42 % had HSV and CMV lung reactivations, respectively; whereas among the 89 influenza patients, 63% and 28% had CMV lung reactivations, respectively. Cumulative incidence of HSV lung reactivation (taking into account extubation and death as competing events) was higher in influenza than in Covid-19 patients (p = 0.03, see figure 1), whereas the rate of HSV bronchopneumonitis was similar in both groups (31% and 25%, respectively). Cumulative incidence of CMV lung reactivation (taking into account extubation and death as competing events) was similar in Covid-19 and influenza patients (p=0.07). Outcomes of patients with HSV or CMV lung reactivations were similar to that of patients without, whatever the underlying conditions, i.e., in Covid-19 patients, in influenza patients, or when all patients were grouped.

Estimated cumulative incidence of herpes simplex virus (HSV) lung reactivation, extubation or death in Covid-19 and influenza patients, taking into account only the first event that occurred.

**Figure:**
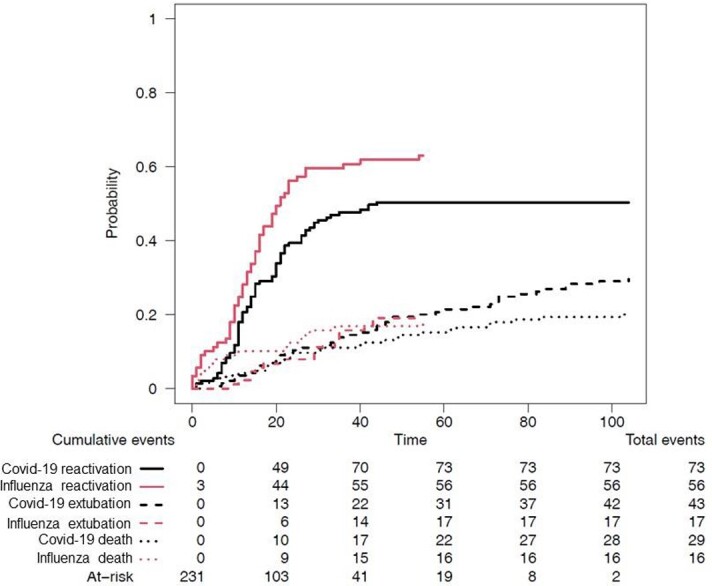

p values for differences between Covid-19 and influenza patients were 0.03 for HSV reactivation, 0.53 for death and 0.87 for extubation.

**Conclusion:**

HSV and CMV lung reactivations are frequent in Covid-19 patients, but not more frequent than in patients with influenza-associated severe pneumonia, despite a higher severity of illness at intensive care unit (ICU) admission of the latter and a longer duration of mechanical ventilation of the former. Although no impact on outcome of HSV and CMV lung reactivations was detected, the effect of antiviral treatment against these *Herpesviridae* remains to be determined in these patients.

**Disclosures:**

**CHARLES-EDOUARD LUYT, MD PhD**, AdvanzPharma: Honoraria|Aerogen: Honoraria|Merck: Honoraria.

